# Exploring the Link Between the Geographical Origin of European Fermented Foods and the Diversity of Their Bacterial Communities: The Case of Fermented Meats

**DOI:** 10.3389/fmicb.2019.02302

**Published:** 2019-10-09

**Authors:** Emiel Van Reckem, Wim Geeraerts, Christina Charmpi, David Van der Veken, Luc De Vuyst, Frédéric Leroy

**Affiliations:** Research Group of Industrial Microbiology and Food Biotechnology (IMDO), Faculty of Sciences and Bioengineering Sciences, Vrije Universiteit Brussel, Brussels, Belgium

**Keywords:** meat fermentation, meat microbiota, European fermented meat products, geographical origin, starter cultures

## Abstract

European fermented meat products are prepared according to a wide variety of different recipes and processing conditions, which can influence their fermentative microbiota. However, due to the diverse processing conditions applied across Europe, it remained unclear to which degree bacterial heterogeneity can be encountered in commercially available fermented meat products and whether this is linked to their geographical origin. Therefore, the bacterial species diversity of 80 fermented meat products available in the Belgian retail, coming from five different countries, was investigated. It was also assessed how this related to the country of origin and the key processing parameters pH and salt concentration. The samples originated from Belgium, France, Germany, Italy, and Spain. In general, Southern European fermented meat products commonly had a higher pH, with their lactic acid bacteria (LAB) communities being represented by *Lactobacillus sakei* and with mostly *Staphylococcus xylosus* and *Staphylococcus equorum* governing over the coagulase-negative staphylococci (CNS) communities. Among these products, the Spanish variants showed a higher prevalence of *S. equorum*, whereas *S. xylosus* was the prevailing CNS species in most French and Italian fermented meat products. In contrast, Northern European fermented meat products were generally more acidified and showed a higher prevalence of *Pediococcus pentosaceus* in their LAB communities, whereas *Staphylococcus carnosus* represented the CNS communities. Non-parametric statistical tests indicated the impact of the geographical origin on the prevalence of the LAB and CNS species. The latter was likely due to the combination of differences in process technology as well as starter culture use.

## Introduction

Storytelling is frequently used to enhance the cultural meaning of fermented foods (Leroy et al., 2015). This includes references to geographical origin, thus accentuating authenticity and the local technological specificities that suggest artisanship and tradition (Grunert, 2006; Leroy et al., 2013; Fenger et al., 2015). For instance, the production of lambic sour beers is said to be aided, not only by the local climate as well as by inoculation with a specific air microbiota of the Senne river valley, an area near Brussels (Spitaels et al., 2014). Microbiome compositions can be used to predict the geographical origin of grapes, whereby regional variation is driven by such factors as climate, soil, and plant cultivar (Bokulich et al., 2014; Mezzasalma et al., 2018). Another example is sourdough bread, whose numerous regional variants, characterized by diverse microbial compositions, are available and believed to depend on ingredients traditionally associated with local culture and origin (De Vuyst et al., 2017; Palla et al., 2017). Such relationships are not necessarily clear-cut, as upon closer scrutiny such claimed region-dependent idiosyncrasies often appear to be artifacts resulting from study design or interpretation of the research data (De Vuyst et al., 2017; Van Kerrebroeck et al., 2017). Moreover, the investigation of regional differentiation is often coarse and stereotyped. Nonetheless, there is a clear opportunity to better exploit the potential of the “virtuous” microbial diversity of a given *terroir* or specific production method within the overall framework of origin denomination (Capozzi and Spano, 2011; Capozzi et al., 2012). This is of particular importance as diversity – even below species level – has the potential to affect the metabolome and, therefore, quality and typicity (Montanari et al., 2018).

In the case of European fermented meats, a binary differentiation between the North and South of Europe is usually reported (Leroy et al., 2006). North-European fermented sausages tend to be acid (pH of 5.0 or lower) and are fermented more rapidly and at higher temperatures than Mediterranean-type sausages (Holck et al., 2015). The latter are prepared using a slower acidification process and are more extensively dried and ripened (Hierro et al., 2015). Additionally, South-European fermented sausages are often heavily spiced and sometimes overgrown with desirable molds, whereas a smoking step is commonly applied in Northern Europe to inhibit mold growth (Janssens et al., 2013; Leroy et al., 2013). It is fair to assume that such pronounced differences in processing conditions may influence desirable bacterial growth and species diversity, in particular with respect to prevailing fractions of lactic acid bacteria (LAB) and catalase-positive cocci, including the coagulase-negative staphylococci (CNS). In spontaneously fermented sausages, *Lactobacillus sakei* is frequently encountered as the most prevalent species, followed by *Lactobacillus curvatus* and *Lactobacillus plantarum* that occur sometimes in Southern European sausages (Drosinos et al., 2005; Rantsiou et al., 2005; Janssens et al., 2012; Cocconcelli and Fontana, 2015). Among CNS, a far greater species diversity is found, although *Staphylococcus xylosus, Staphylococcus equorum*, and *Staphylococcus saprophyticus* are usually the most reported species (Drosinos et al., 2005; Coton et al., 2010; Bermúdez et al., 2012; Greppi et al., 2015; Sánchez Mainar et al., 2017). However, outcomes are confounded by the fact that fermented sausage production is nowadays usually initiated by the addition of a starter culture. In Europe, *L. sakei* is predominantly used as a LAB starter culture (Cocconcelli, 2007; Ojha et al., 2015), whilst *S. xylosus* and *Staphylococcus carnosus* can be used separately or in combination for the inoculation of CNS (Ojha et al., 2015; Stavropoulou et al., 2018b). Whether such starter cultures are always able to overrule the natural microbiota is doubtful, given the wide variety of processing conditions applied around Europe (Leroy et al., 2006; Sawitzki et al., 2009). Indeed, previous analyses have indicated that this is not necessarily the case, with the pH variation being a major factor of influence (Stavropoulou et al., 2018a).

The aim of the present study was to get an overview of the bacterial species diversity within fermented meat products available in (and representative for) the Belgian retail and to assess the relationship with their country of origin and two of the key processing parameters, namely pH and salt concentration.

## Materials and Methods

### Sampling and Experimental Set-Up

A total of 80 randomly selected fermented meat products was purchased from the Belgian retail with 44 products originating from local markets and small retail vendors and 36 products originating from major supermarket chains in and around Brussels. For the supermarkets in particular, the sampling aimed at being exhaustive as to be representative for common purchases and to include all items with the highest sales volumes in Belgian retail. For each fermented meat product, the label was analyzed for relevant information, in particular the salt concentration present (explicitly mentioned on 50 out of 80 fermented meat products) and the country of production (Belgium, France, Germany, Italy, and Spain).

### Enumeration and Isolation of Microorganisms

For bacterial enumeration, 12 g of each fermented meat product were aseptically transferred into a stomacher bag (Seward, Worthing, West Sussex, United Kingdom) and mixed with 108 ml of recovery diluents [sterile solution of 0.85% (m/v) NaCl (VWR International, Darmstadt, Germany) and 0.1% (m/v) bacteriological peptone (Oxoid, Basingstoke, Hampshire, United Kingdom)]. The mixture was made at maximum speed for 2.5 min in a Laboratory Blender Stomacher 400 (Seward). Appropriate serial decimal dilutions in saline were prepared. Subsequently, these dilutions were spread on mannitol-salt-phenol-red agar (MSA; VWR International) and de Man-Rogosa-Sharpe (MRS) agar (Oxoid) for enumeration of presumptive CNS and LAB, respectively, after which the agar media were incubated at 30°C for 72 h. Thereafter, agar media containing 30–300 colonies were used for determining the bacterial counts and 5–30% of the colonies present were randomly selected and picked up to follow the bacterial community dynamics. The colonies picked up from the MSA and MRS agar media were transferred into brain heart infusion (BHI; Oxoid) medium and incubated overnight at 30°C to acquire grown cultures to be used for DNA extraction as well as storage at −80°C in cryovials containing 25% (v/v) of glycerol.

### pH Measurement

After sampling, the pH was measured directly in the meat batter with a DY-P10 pH meter (Sartorius, Göttingen, Germany), equipped with an insertion pH probe (VWR International). Three independent measurements were performed per sample.

### Classification and Identification of Bacterial Isolates Through (GTG)_5_-PCR Fingerprinting of Genomic DNA

Genomic DNA extraction from cell pellets, procured by microcentrifugation at 13,000 rpm of 1.5 ml of an overnight culture of the isolates mentioned above, was performed with a Nucleospin 96 tissue kit (Macherey Nagel, Düren, Germany), according to the manufacturer’s instructions. Ahead of extraction, all cell pellets were washed with Tris-ethylene diaminotetraacetic acid (EDTA)-sucrose buffer [TES buffer; 50 mM Tris base (Calbiochem, Darmstadt, Germany), 1 mM EDTA (Sigma-Aldrich, St. Louis, MO, United States), and 6.7% (m/v) sucrose (VWR International); pH 8.0]. Subsequently, (GTG)_5_-PCR fingerprints of the genomic DNA were generated, followed by image analysis, as previously described (Braem et al., 2011). Numerical analysis of the fingerprints obtained was performed with BioNumerics 5.1 software (Applied Maths, Sint-Martens-Latem, Belgium). Confirmation of the species identity assigned to each cluster was done by sequencing of the 16S rRNA for LAB and/or *rpoB* and *tuf* genes for CNS of representative isolates, as previously described (Heikens et al., 2005; Braem et al., 2011). For the molecular distinction between *L. sakei* and *L. curvatus*, the reverse primers Ls (5′-ATG AAA CTA TTA AAT TGG TAC-3′) and Lc (5′-TTG GTA CTA TTT AAT TCT TAG-3′), coupled with the forward primer 16S (5′-GCT GGA TCA CCT CCT TTC-3′), were used (Berthier and Ehrlich, 1998; Urso et al., 2006). The accession numbers of the genes used for identification of the representative cluster isolates, as well as their percentages of identity, are listed in Table 1.

**TABLE 1:** Identities (%) of the sequenced genes (16S rRNA, *rpoB*, and/or *tuf*) of representative (GTG)_5_-PCR fingerprint cluster isolates from MRS agar and MSA and the accession numbers of the entries with the highest identity.

**Gene**	**Accession number**	**%**	**Species**
16S	CP018867.1	99	*Lactobacillus alimentarius*
16S	NR_114915.1	100	*Lactobacillus curvatus*
16S	NR_104573.1	99	*Lactobacillus plantarum*
16S	NR_113338.1	99	*Lactobacillus plantarum*
16S	NR_113821.1	99	*Lactobacillus sakei*
16S	NR_115172.1	99	*Lactobacillus sakei*
16S	NR_042058.1	99	*Pediococcus pentosaceus*
*tuf*	CP016760.1	100	*Staphylococcus carnosus*
*tuf*	CP012968.1	99	*Staphylococcus equorum*
*rpoB*	CP013714.1	99	*Staphylococcus equorum*
*tuf*	CP013980.1	100	*Staphylococcus equorum*
*rpoB*	CP013980.1	100	*Staphylococcus equorum*
*tuf*	CP022056.2	99	*Staphylococcus saprophyticus*
*rpoB*	CP022093.1	99	*Staphylococcus saprophyticus*
*tuf*	CP018199.1	99	*Staphylococcus succinus*
*rpoB*	CP018199.1	98	*Staphylococcus succinus*
*tuf*	CP008724.1	100	*Staphylococcus xylosus*
*tuf*	CP013922.1	99	*Staphylococcus xylosus*
*rpoB*	LN554884.1	99	*Staphylococcus xylosus*

### Statistics

Intra-sample diversity (alpha-diversity) was assessed by calculating the Simpson (diversity) and Pielou (evenness) indexes. Inter-sample diversity (beta-diversity) to determine differences in bacterial composition between fermented meat products produced in different European countries (Belgium, France, Germany, Italy, and Spain) was assessed by conducting a permutational multivariate analysis of variance (PERMANOVA), based on Bray–Curtis dissimilarity scores. This analysis was followed by a series of pairwise PERMANOVA comparisons and a similarity percentage analysis (SIMPER) to assess the bacterial differences between fermented meat products produced in the countries mentioned above. The vegan (version 2.5-4, Oksanen et al., 2019) and RVAideMemoire packages (version 0.9-73, Hervé, 2019) were implemented for both intra-sample and inter-sample variability. All samples were also subjected to a detrended correspondence analysis (DCA) (Hill and Gauch, 1980), as to visualize species distribution patterns. DCA was performed using vegan and visualized by using the package ggplot2 (version 3.1.1, Wickham, 2016). Subsequently, one-way analyses of variance (ANOVAs) were conducted for the determination of differences in bacterial enumerations, pH, and salt concentrations between fermented meat products produced in the various countries, followed by a series of *post hoc* pairwise comparisons with Tukey’s test. A threshold value of 0.05 was considered to be significant for all statistical procedures applied. All statistical analyses and tests performed were executed through the RStudio software (version 3.5.2; R Core Team, 2018).

## Results

### Differences in pH, Salt Concentration, and Bacterial Counts According to the Country of Origin

Out of the 80 samples of fermented meat products examined, 13 products originated from Belgium (coming from at least nine different producers), 32 products from France (at least 21 different producers), four products from Germany (four different producers), 17 products from Italy (at least 12 different producers), and 14 products from Spain (at least 11 different producers). In 50 of these 80 samples, the salt concentration was explicitly mentioned on the label. In this subset of 50 samples, 10 products originated from Belgium, 16 products from France, 3 products from Germany, 11 products from Italy, and 9 products from Spain. The average salt concentration amounted to 3.5 ± 0.5, 4.8 ± 0.5, 3.8 ± 0.0, 3.9 ± 0.5, and 3.6 ± 0.5%, respectively. The salt concentration in French fermented meat products was shown to be significantly higher (*P* < 0.05) than in fermented meat products originating from other parts of Europe.

**FIGURE 1:**
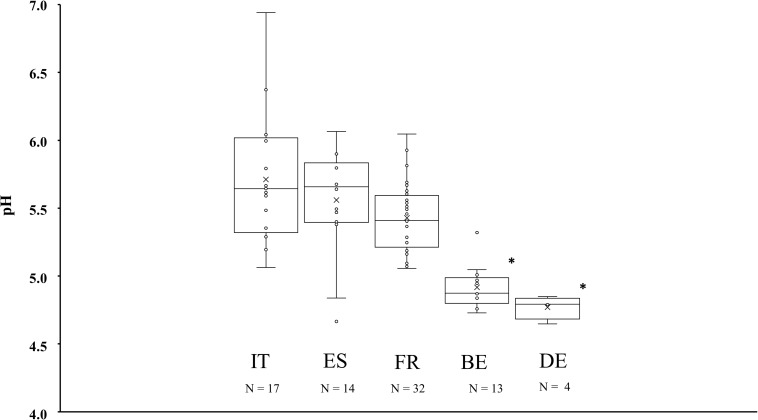
Boxplots of the average pH values measured in fermented meat products originating from BE (Belgium), DE (Germany), ES (Spain), FR (France), and IT (Italy). Significant differences are indicated with an asterisk.

Differences in pH were found according to the country of origin (Figure 1). In decreasing order, the average pH was 5.71 ± 0.47, 5.56 ± 0.40, 5.43 ± 0.25, 4.92 ± 0.16, and 4.77 ± 0.09 for Italian, Spanish, French, Belgian, and German fermented meat products, respectively. Fermented sausages produced in Belgium and Germany had a significantly lower pH (*P* < 0.05) than fermented meat products produced in Italy, Spain, and France. No significant differences were found between the pH of fermented meat products from Belgium and Germany, or between those from Italy, Spain, and France. Nevertheless, some atypical cases were found among the Southern European fermented meat products. Samples TM79 and TM86 originating from Italy, samples TM54 and TM84 from Spain, and samples TM19, TM58, TM65, TM66, TM69, and TM102 from France each had a pH lower than 5.2, therefore being more comparable to the values encountered in Northern European fermented meat products.

**FIGURE 2:**
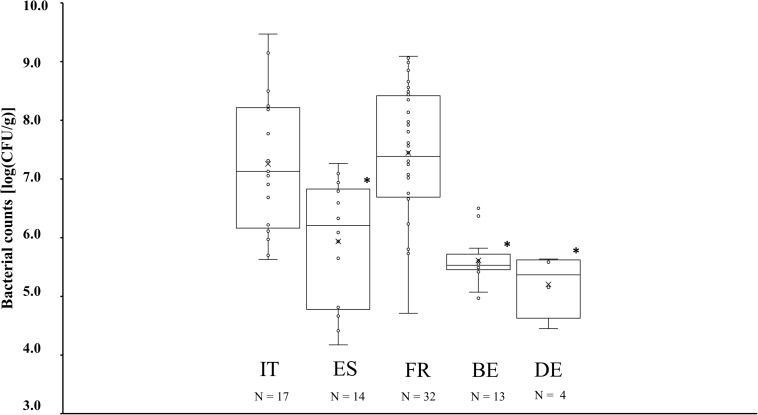
Boxplots of the bacterial counts on mannitol-salt-agar (MSA) media, expressed in log (cfu/g), for fermented meat products originating from BE (Belgium), DE (Germany), ES (Spain), FR (France), and IT (Italy). Significant differences are indicated with an asterisk.

Presumable CNS counts derived from plating on MSA media varied across the different producing countries (Figure 2). MSA counts in fermented meat products originating from Italy and France, amounting to 7.3 ± 1.2 log (CFU/g) and 7.4 ± 1.1 log (CFU/g), respectively, were significantly higher (*P* < 0.05) than MSA counts in products from Spain, Belgium, and Germany, which amounted to 5.9 ± 1.0 log (CFU/g), 5.6 ± 0.4 log (CFU/g), and 5.2 ± 0.5 log (CFU/g), respectively. However, some deviating cases were noticed. Among the Italian and French fermented meat products, MSA counts in samples TM52, TM58, TM65, TM79, and TM83 remained below 6.0 log (CFU/g), whereas in the Spanish product samples TM56 and TM104, the MSA counts amounted to more than 7.0 log (CFU/g) (Supplementary Table S1). Presumable LAB counts derived from the MRS agar media were more similar across all fermented meat products. MRS counts equaled to 7.9 ± 0.5 log (CFU/g), 7.8 ± 1.1 log (CFU/g), 8.1 ± 0.5 log (CFU/g), 8.1 ± 0.3 log (CFU/g), and 8.1 ± 0.5 log (CFU/g) for products produced in Italy, Spain, France, Belgium, and Germany, respectively, and did not show any significant differences.

### Species Diversity of CNS and LAB Communities in Fermented Meat Products According to the Country of Origin

**FIGURE 3:**
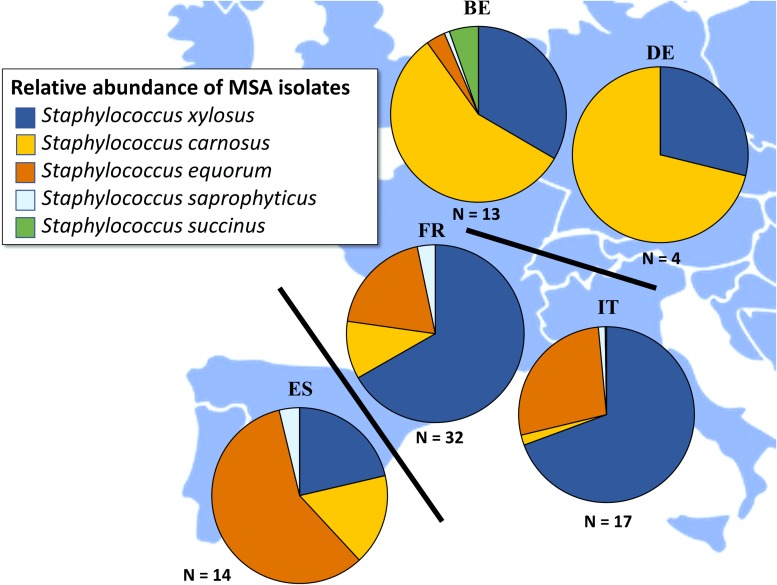
Total relative abundances of *Staphylococcus carnosus* (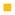), *Staphylococcus equorum* (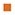), *Staphylococcus saprophyticus* (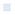), *Staphylococcus succinus* (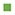), and *Staphylococcus xylosus* (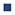) in fermented meat products originating from BE (Belgium), DE (Germany), ES (Spain), FR (France), and IT (Italy). Isolates were obtained from mannitol-salt-agar (MSA) media.

In general, across all fermented meat products and countries of origin, the following five CNS species were encountered: *S. carnosus, S. equorum*, *S. saprophyticus*, *Staphylococcus succinus*, and *S. xylosus* (Figure 3). In Italian fermented meat products, *S. xylosus* was the prevalent CNS species, followed by *S. equorum* and the occasional presence of *S. saprophyticus* and *S. carnosus*. Notable exceptions were samples TM79, in which *S. saprophyticus* was the prevailing CNS species, and samples TM83 and TM86, in which *S. carnosus* was the prevalent one (Supplementary Figure S1). CNS communities in Spanish fermented sausages were mainly represented by *S. equorum*, followed by *S. xylosus* and *S. carnosus*, with *S. saprophyticus* occurring as a minor species. Noteworthy is that only one Spanish fermented meat product, sample TM54, was completely represented by *S. carnosus* (Supplementary Figure S2). Fermented meat products originating from France were represented by *S. xylosus*, in most cases accompanied by *S. equorum* (Supplementary Figure S3). The species *S. carnosus*, *S. saprophyticus*, and *S. succinus* occurred as subdominant fractions. In Belgian and German fermented meat products, *S. carnosus* was generally the prevailing CNS species, followed by *S. xylosus* and, to a lesser degree, by *S. equorum*, *S. saprophyticus*, and *S. succinus* in some samples. *S. succinus* was encountered as the prevailing CNS species in only one Belgian fermented meat product, namely sample TM67 (Supplementary Figure S4).

**FIGURE 4:**
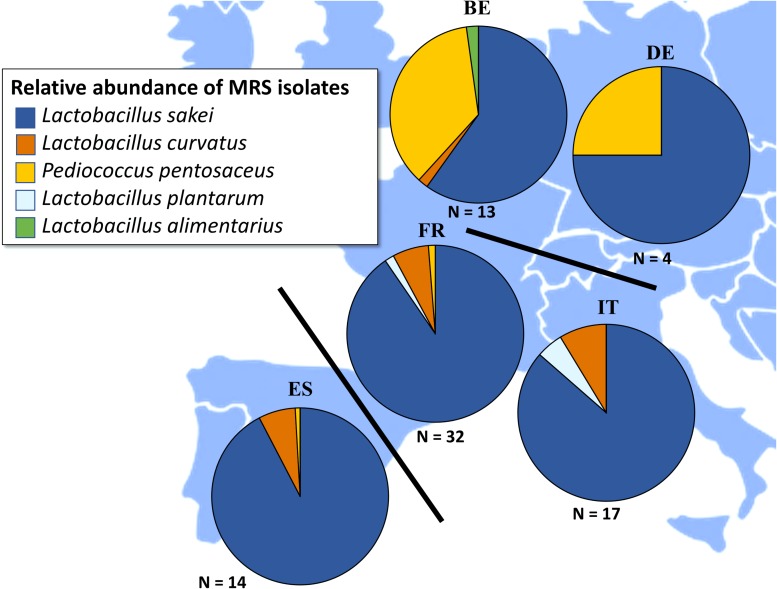
Total relative abundances of *Lactobacillus alimentarius* (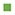), *Lactobacillus curvatus* (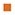), *Lactobacillus plantarum* (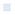), *Lactobacillus sakei* (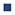), and *Pediococcus pentosaceus* (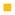) in fermented meat products originating from BE (Belgium), DE (Germany), ES (Spain), FR (France), and IT (Italy). Isolates were obtained from de Man-Rogosa-Sharpe (MRS) agar media.

Considering the LAB communities, five different species were encountered, i.e., *L. sakei*, *L. curvatus*, *L. plantarum*, *Lactobacillus alimentarius*, and *Pediococcus pentosaceus* (Figure 4). *L. sakei* was by far the prevailing LAB species in the fermented meat products originating from France, Italy, and Spain, with *L. curvatus*, *L. plantarum*, *L. alimentarius*, and *P. pentosaceus* only occurring sporadically. *L. curvatus* was encountered as the prevailing LAB species in only three samples, namely TM27, TM52, and TM93, whereas *L. plantarum* was only found once as the prevailing LAB species, namely in sample TM79 (Supplementary Figures S5–S7). Although *L. sakei* was still frequently the prevailing LAB species in Belgian and German fermented meat products as well, *P. pentosaceus* was often found as prevailing LAB species, namely in samples TM70, TM71, TM72, TM77, TM81, and TM82 (Supplementary Figure S8).

### Alpha- and Beta-Diversity

**FIGURE 5:**
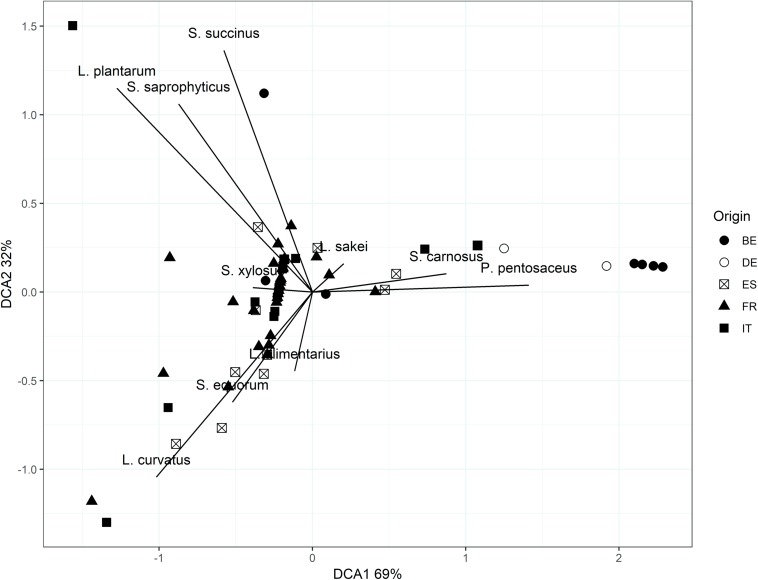
Detrended correspondence analysis (DCA) biplot based on Bray–Curtis dissimilarity scores of the microbial communities of fermented meat products originating from BE (Belgium), DE (Germany), ES (Spain), FR (France), and IT (Italy).

The bacterial diversity was relatively limited in all fermented meat products examined, no matter where they were produced (Table 2). However, based on a PERMANOVA, the microbial communities of the fermented meat products originating from different countries were shown to be statistically different (*P* < 0.05). This could also be seen, to some extent, in the visualization of the beta-diversity using DCA (Figure 5). Samples derived from the same country grouped together, indicating similar bacterial compositions. Pairwise PERMANOVA tests showed that the bacterial profiles in fermented meat products produced in Belgium and Germany were significantly different (*P* < 0.05) from the bacterial profiles of products from France, Italy, and Spain. Yet, the microbial communities in fermented meat products coming from Spain were in turn also significantly different (*P* < 0.05) from the communities found in French and Italian products. No significant differences were found between the microbial communities in German and Belgian fermented meat products or between French and Italian ones. A SIMPER analysis showed that differences between the Northern-European (i.e., Belgian and German fermented meat products) and the Southern-European groups (i.e., French, Italian, and Spanish fermented meat products) were mainly due to differences in the prevalence of *S. carnosus*, *S. xylosus*, *S. equorum*, and, to a lesser extent, *L. sakei* and *P. pentosaceus*. Differences between the Spanish fermented meat products and the cluster of French and Italian ones could be mainly ascribed to the variations in the presence of *S. equorum* and *S. xylosus*.

**TABLE 2:** Alpha-diversity metrics based on the relative abundances of bacterial species found through (GTG)_5_-PCR fingerprinting of genomic DNA.

**Sample code**	**Simpson (D)**	**Pielou (Je)**	**Country of origin**
TM31	0.50	1.00	BE
TM34	0.66	0.89	BE
TM46	0.50	1.00	BE
TM64	0.55	0.78	BE
TM67	0.61	0.91	BE
TM70	0.52	0.72	BE
TM71	0.50	1.00	BE
TM72	0.55	0.79	BE
TM73	0.50	1.00	BE
TM74	0.50	1.00	BE
TM77	0.50	1.00	BE
TM82	0.57	0.83	BE
TM60	0.56	0.82	DE
TM62	0.50	1.00	DE
TM76	0.50	1.00	DE
TM81	0.63	0.80	DE
TM10	0.66	0.87	ES
TM16	0.57	0.84	ES
TM35	0.78	0.88	ES
TM44	0.66	0.85	ES
TM50	0.50	1.00	ES
TM54	0.50	1.00	ES
TM56	0.50	1.00	ES
TM57	0.60	0.75	ES
TM59	0.64	0.84	ES
TM84	0.60	0.74	ES
TM85	0.50	1.00	ES
TM88	0.64	0.84	ES
TM91	0.50	1.00	ES
TM103	0.74	0.91	ES
TM104	0.50	1.00	ES
TM13	0.57	0.84	FR
TM15	0.64	0.82	FR
TM18	0.60	0.90	FR
TM19	0.61	0.92	FR
TM20	0.57	0.84	FR
TM21	0.50	1.00	FR
TM22	0.54	0.76	FR
TM23	0.64	0.75	FR
TM24	0.61	0.92	FR
TM25	0.58	0.86	FR
TM26	0.57	0.85	FR
TM28	0.57	0.84	FR
TM29	0.61	0.91	FR
TM39	0.64	0.85	FR
TM41	0.54	0.76	FR
TM45	0.57	0.84	FR
TM52	0.59	0.89	FR
TM55	0.53	0.75	FR
TM58	0.73	0.98	FR
TM65	0.62	0.78	FR
TM66	0.54	0.77	FR
TM69	0.60	0.68	FR
TM80	0.50	1.00	FR
TM90	0.74	0.90	FR
TM92	0.73	0.89	FR
TM95	0.57	0.85	FR
TM96	0.54	0.75	FR
TM98	0.61	0.92	FR
TM99	0.59	0.88	FR
TM100	0.62	0.93	FR
TM101	0.59	0.89	FR
TM102	0.69	0.91	FR
TM2	0.60	0.90	IT
TM7	0.53	0.74	IT
TM12	0.63	0.95	IT
TM27	0.67	0.87	IT
TM30	0.61	0.92	IT
TM32	0.59	0.89	IT
TM33	0.54	0.76	IT
TM37	0.62	0.94	IT
TM47	0.50	1.00	IT
TM61	0.50	1.00	IT
TM63	0.50	1.00	IT
TM79	0.72	0.95	IT
TM83	0.60	0.90	IT
TM86	0.50	1.00	IT
TM87	0.53	0.73	IT
TM89	0.66	0.85	IT
TM93	0.69	0.91	IT

*The Simpson (D) and Pielou (Je) indexes were calculated for all the samples to measure their diversity and evenness, respectively. Samples are ordered according to their country of origin, which are indicated as BE (Belgium), DE (Germany), ES (Spain), FR (France), and IT (Italy).*

## Discussion

Differences in geographical origin affect both the perceptions and attributes of fermented foods (Spitaels et al., 2014; Fenger et al., 2015; Palla et al., 2017). This is also the case for fermented meat products, of which many different variants are produced, particularly across Europe (Leroy et al., 2015; Ojha et al., 2015). The use of Protected Designation of Origin (PDO) and Protected Geographical Indication (PGI) labels further illustrates this point (Leroy et al., 2015). In the category of meat products of the DOOR list of the European Union^1^, there are 36 and 146 products awarded with PDO and PGI labels, respectively, as by June 2019.

When comparing fermented meat products from different countries across Europe, variations in technological properties were found, based on label analysis (salt) and pH measurements. Although salt levels were roughly equivalent (3–4%) throughout Europe, products from France were more heavily salted, with an average concentration of 4.8% and ranging from 3.8 to 5.4%. Generally, salt concentrations in cured meats are known to vary within and among European countries, usually between 2 and 6%, which has been attributed to a lack of specific guidelines and, hence, traditional practices (Jiménez-Colmenero et al., 2001; Newson et al., 2013; Delgado-Pando et al., 2018). A lower limit for microbial safety and sensory quality has been identified at about 2% of salt (Desmond, 2006). Despite an ongoing trend for salt reduction (Desmond, 2006; Toldrá and Reig, 2011), the fermented meat products that were included in the present study to reflect the market variability in Belgium displayed relatively conventional salt levels.

The most acidified fermented meat products originated from Belgium and Germany, with pH values below 5.0. This showcases the differences in acidification between Northern and Southern European fermented meat products, with the latter being less acidic than the former (Janssens et al., 2013; Hierro et al., 2015; Holck et al., 2015). Fermented sausages produced in Belgium and Germany thus matched the overall description of Northern European products based on their acidity levels, whereas fermented sausages produced in France, Italy, and Spain were more akin to the Southern European prototype.

Regarding bacterial species diversity, the CNS communities of French and Italian fermented meat products were represented by *S. xylosus*, with *S. equorum* being the second most prevalent CNS species. Spanish fermented meat products, however, were mainly represented by *S. equorum*. This corroborated with previous studies, during which *S. xylosus* and *S. equorum* typify Southern European fermented meat products (Blaiotta et al., 2004; Baruzzi et al., 2006: Iacumin et al., 2006; Fontán et al., 2007; Martín et al., 2007; Fonseca et al., 2013; Greppi et al., 2015). Both species seem to prefer meat fermentations at relatively high pH (i.e., above 5.3) and lower temperatures, which are conditions often applied during Southern European fermentation processes (Janssens et al., 2013; Stavropoulou et al., 2018c). Although *S. saprophyticus* can also be encountered in Southern European products (Drosinos et al., 2005; Pisacane et al., 2015), this species was only encountered occasionally in the present study and as a subdominant one. This might be explained by the relatively high pH, favoring the presence of *S. xylosus* and *S. equorum* over the more acid-adapted *S. saprophyticus* (Janssens et al., 2013; Stavropoulou et al., 2018c). This is further illustrated by the fact that *S. saprophyticus* was only found once as prevailing CNS species, namely in an Italian fermented sausage (sample TM79) that was characterized by a rather low pH of 5.2. Typically, Belgian and German fermented meat products showed a higher prevalence of *S. carnosus*. This was in line with previous observations, during which *S. carnosus* prevails as an acid-tolerant CNS species in the more acidic meat fermentation processes of the Northern European type (Stahnke et al., 2002; Ravyts et al., 2010; Janssens et al., 2012; Aquilanti et al., 2016; Stavropoulou et al., 2018c). This acid-tolerant characteristic was also confirmed in the few Spanish and Italian fermented meat products, in which *S. carnosus* was the prevailing CNS species (samples TM54, TM83, and TM86), that all were more acidified than their respective averages. Since *S. carnosus* is seldomly prevailing during spontaneous fermentation processes and is regularly applied as a meat starter culture, it is likely that the fermented meat products in which it was encountered were produced using starter cultures (Talon et al., 2007; Ravyts et al., 2012). Yet, whether or not a starter culture was applied, the CNS counts in Northern European fermented meat products were generally lower than those encountered in Italian and French ones. This might be due to the lower pH of the Northern European products, which generally reduces growth of staphylococci (Søndergaard and Stahnke, 2002). The latter is also confirmed by those Italian and French products in which both a low pH and low CNS counts were found (samples TM58, TM65, and TM79). The lower CNS counts in Spanish fermented meat products compared to Italian and French ones might parallel the presence of *S. equorum* as the prevailing CNS species. In contrast to *S. xylosus*, *S. equorum* is generally not added as a starter culture, suggesting the absence of external bacteria and, therefore, a lower initial microbial load and growth (Ojha et al., 2015).

The LAB communities in the present study were less diverse than the CNS ones, with *L. sakei* as the prevailing LAB species in most fermented meat products, regardless of the country of origin. The LAB species diversity of fermented meats is known to be limited, with *L. sakei* usually prevailing during most European meat fermentation processes, due to its excellent adaptation to and competitiveness in the meat matrix (Chaillou et al., 2005; Janssens et al., 2013; Aquilanti et al., 2016; Montanari et al., 2018; Stavropoulou et al., 2018c). In addition, *L. plantarum, L. curvatus*, and *P. pentosaceus* were occasionally encountered, mostly as subdominant species. Although less common, these species are indeed not alien to fermented meats (Aquilanti et al., 2016; Stavropoulou et al., 2018c). In several fermented meat products produced in Belgium and Germany, *P. pentosaceus* was even found as the prevailing LAB species. This may be explained by the fact that *P. pentosaceus* is sometimes used as a starter culture (together with glucono-δ-lactone) instead of (or in addition to) *L. sakei* or *L. curvatus* to accelerate the acidification, although mainly in Netherlands and the United States where higher fermentation temperatures are applied (Leroy et al., 2006; Ravyts et al., 2012; Kumar et al., 2017). Whenever *P. pentosaceus* was found as the prevailing LAB species, the prevailing species among the CNS was *S. carnosus*, which is also known for its acid tolerance and use in starter cultures (Janssens et al., 2012; Ojha et al., 2015; Aquilanti et al., 2016; Stavropoulou et al., 2018c).

Overall, the present study showed that fermented meat products produced in Northern and Southern Europe, and distributed in the Belgian retail, cluster into distinct groups. Southern European fermented meat products generally had a higher pH and their LAB and CNS communities were represented by *L. sakei, S. xylosus*, and *S. equorum*, respectively. Among Southern European fermented meat products, Spanish ones showed a pre-eminence of *S. equorum*, whereas French and Italian ones were mainly represented by *S. xylosus*. In contrast, Northern European fermented meat products had a low pH, which was reflected in the composition of their LAB and CNS communities. Generally, these communities showed a higher relative abundance of *P. pentosaceus* as LAB species and were governed by *S. carnosus* as CNS species. In conclusion, despite the existence of some in-group variability, the geographical origin of fermented meat products did indeed translate in a certain level of microbial typicity. The latter was likely due to the combination of differences in process technology as well as starter culture use. Future research will have to identify how such variability translates into consumer preferences in the Belgian market and beyond. A comparison with fermented meats from other continents, encompassing a myriad of ethnic products, could not only yield additional microbial and ecological insights but also underscore the socio-cultural benefits of food heritage (Tamang et al., 2016).

## Data Availability Statement

All datasets generated for this study are included in the manuscript/Supplementary Files.

## Author Contributions

FL, WG, and ER contributed to the design of the study. CC, DV, ER, and WG contributed to the sample collection and performed the bacterial analysis. ER performed the statistical analysis and wrote the manuscript. FL and LV contributed to the analysis or interpretation of the data. All authors revised and approved the submitted version.

## Conflict of Interest

The authors declare that the research was conducted in the absence of any commercial or financial relationships that could be construed as a potential conflict of interest.

## Footnotes

1 https://ec.europa.eu/agriculture/quality/door/list.html

## References

[B1] Aquilanti L. Garofalo C. Osimani A. Clementi F. (2016). Ecology of lactic acid bacteria and coagulase-negative cocci in fermented dry sausages manufactured in Italy and other Mediterranean countries: an overview. *Int. Food Res. J.* 23 429–445.

[B2] Baruzzi F. Matarante A. Caputo L. Morea M. (2006). Molecular and physiological characterization of natural microbial communities isolated from a traditional Southern Italian processed sausage. *Meat Sci.* 72 261–269. 10.1016/j.meatsci.2005.07.013 22061553

[B3] Bermúdez R. Lorenzo J. M. Fonseca S. Franco I. Carballo J. (2012). Strains of *Staphylococcus* and *Bacillus* isolated from traditional sausages as producers of biogenic amines. *Front. Microbiol.* 3:151. 10.3389/fmicb.2012.00151 22529847 PMC3328851

[B4] Berthier F. Ehrlich S. D. (1998). Rapid species identification within two groups of closely related lactobacilli using PCR primers that target the 16S/23S rRNA spacer region. *FEMS Microbiol. Lett.* 161 97–106. 10.1016/s0378-1097(98)00055-x 9561736

[B5] Blaiotta G. Pennacchia C. Villani F. Ricciardi A. Tofalo R. Parente E. (2004). Diversity and dynamics of communities of coagulase-negative staphylococci in traditional fermented sausages. *J. Appl. Microbiol.* 97 271–284. 10.1111/j.1365-2672.2004.02298.x 15239693

[B6] Bokulich N. A. Thorngate J. H. Richardson P. M. Mills D. A. (2014). Microbial biogeography of wine grapes is conditioned by cultivar, vintage, and climate. *Proc. Natl. Acad. Sci. U.S.A.* 111 E139–E148. 10.1073/pnas.1317377110 24277822 PMC3890796

[B7] Braem G. De Vliegher S. Supré K. Haesebrouck F. Leroy F. De Vuyst L. (2011). (GTG)5-PCR fingerprinting for the classification and identification of coagulase-negative *Staphylococcus* species from bovine milk and teat apices: a comparison of type strains and field isolates. *Vet. Microbiol.* 147 67–74. 10.1016/j.vetmic.2010.05.044 20599332

[B8] Capozzi V. Russo P. Spano G. (2012). Microbial information regimen in EU geographical indications. *World Pat. Inf.* 34 229–231. 10.1016/j.wpi.2012.04.001

[B9] Capozzi V. Spano G. (2011). Food microbial biodiversity and “microbes of protected origin”. *Front. Microbiol.* 2:237 10.3389/fmicb.2011.00237 PMC3226094 22144978

[B10] Chaillou S. Champomier-Vergès M. C. Cornet M. Crutz-Le Coq A. M. Dudez A. M. Martin V. (2005). The complete genome sequence of the meat-borne lactic acid bacterium *Lactobacillus sakei* 23K. *Nat. Biotechnol.* 23 1527–1533. 10.1038/nbt1160 16273110

[B11] Cocconcelli P. S. (2007). “Starter cultures: bacteria,” in *Handbook of Fermented Meat and Poultry*, eds Toldrà F. Hui Y. H. Astiasarán I. Nip W. K. Sebranek J. G. Silveira E. T. F. (Ames, IA: Blackwell Publishing), 137–145. 10.1002/9780470376430.ch13

[B12] Cocconcelli P. S. Fontana C. (2015). “Bacteria,” in *Handbook of Fermented Meat and Poultry*, 2nd Edn, eds Toldrà F. Hui Y. H. Astiasarán I. Sebranek J. G. Talon R., (Chichester: John Wiley and Sons), 117–128.

[B13] Coton E. Desmonts M. H. Leroy S. Coton M. Jamet E. Christieans S. (2010). Biodiversity of coagulase-negative staphylococci in French cheeses, dry fermented sausages, processing environments and clinical samples. *Int. J. Food Microbiol.* 137 221–229. 10.1016/j.ijfoodmicro.2009.11.023 20061042

[B14] De Vuyst L. Van Kerrebroeck S. Leroy F. (2017). Microbial ecology and process technology of sourdough fermentation. *Adv. Appl. Microbiol.* 100 49–160. 10.1016/bs.aambs.2017.02.003 28732554

[B15] Delgado-Pando G. Fischer E. Allen P. Kerry J. P. O’Sullivan M. Hamill R. M. (2018). Salt content and minimum acceptable levels in whole-muscle cured meat products. *Meat Sci.* 139 179–186. 10.1016/j.meatsci.2018.01.025 29428882

[B16] Desmond E. (2006). Reducing salt: a challenge for the meat industry. *Meat Sci.* 74 188–196. 10.1016/j.meatsci.2006.04.014 22062728

[B17] Drosinos E. H. Mataragas M. Xiraphi N. Moschonas G. Gaitis F. Metaxopoulos J. (2005). Characterization of the microbial flora from a traditional Greek fermented sausage. *Meat Sci.* 69 307–317. 10.1016/j.meatsci.2004.07.012 22062823

[B18] Fenger M. H. Aschemann-Witzel J. Hansen F. Grunert K. G. (2015). Delicious word. Assessing the impact of short storytelling messages on consumer preferences for variations of a new processed meat product. *Food Qual. Prefer.* 41 237–244. 10.1016/j.foodqual.2014.11.016

[B19] Fonseca S. Cachaldora A. Gómez M. Franco I. Carballo J. (2013). Monitoring the bacterial population dynamics during the ripening of galician chorizo, a traditional dry fermented Spanish sausage. *Food Microbiol.* 33 77–84. 10.1016/j.fm.2012.08.015 23122504

[B20] Fontán M. C. G. Lorenzo J. M. Parada A. Franco I. Carballo J. (2007). Microbiological characteristics of “androlla”, a Spanish traditional pork sausage. *Food Microbiol.* 24 52–58. 10.1016/j.fm.2006.03.007 16943094

[B21] Greppi A. Ferrocino I. La Storia A. Rantsiou K. Ercolini D. Cocolin L. (2015). Monitoring of the microbiota of fermented sausages by culture independent rRNA based approaches. *Int. J. Food Microbiol.* 212 67–75. 10.1016/j.ijfoodmicro.2015.01.016 25724303

[B22] Grunert K. G. (2006). Future trends and consumer lifestyles with regard to meat consumption. *Meat Sci.* 74 149–160. 10.1016/j.meatsci.2006.04.016 22062724

[B23] Heikens E. Fleer A. Paauw A. Florijn A. Fluit A. C. (2005). Comparison of genotypic and phenotypic methods for species-level identification of clinical isolates of coagulase-negative staphylococci. *J. Clin. Microbiol.* 43 2286–2290. 10.1128/jcm.43.5.2286-2290.2005 15872257 PMC1153770

[B24] Hervé M. (2019). *RVAideMemoire: Testing and Plotting Procedures for Biostatistics. R package version 0.9-73*. https://CRAN.R-project.org/package=RVAideMemoire (accessed January 14, 2019).

[B25] Hierro E. Fernández M. de la Hoz L. Ordóñez J. A. (2015). “Mediterranean products,” in *Handbook of Fermented Meat and Poultry*, 2nd Edn, eds Toldrà F. Hui Y. H. Astiasarán I. Sebranek J. G. Talon R., (Chichester: John Wiley and Sons), 301–308.

[B26] Hill M. O. Gauch H. G. (1980). Detrended correspondence analysis: an improved ordination technique. *Vegetatio* 42 47–58. 10.1007/978-94-009-9197-2_7

[B27] Holck A. Heir E. Johannessen T. C. Axelsson L. (2015). “Northern European products,” in *Handbook of Fermented Meat and Poultry*, 2nd Edn, eds Toldrà F. Hui Y. H. Astiasarán I. Sebranek J. G. Talon R., (Chichester: John Wiley and Sons), 313–320.

[B28] Iacumin L. Comi G. Cantoni C. Cocolin L. (2006). Ecology and dynamics of coagulase-negative cocci isolated from naturally fermented Italian sausages. *Syst. Appl. Microbiol.* 29 480–486. 10.1016/j.syapm.2005.11.006 16337767

[B29] Janssens M. Myter N. De Vuyst L. Leroy F. (2012). Species diversity and metabolic impact of the microbiota are low in spontaneously acidified belgian sausages with an added starter culture of *Staphylococcus carnosus*. *Food Microbiol.* 29 167–177. 10.1016/j.fm.2011.07.005 22202870

[B30] Janssens M. Myter N. De Vuyst L. Leroy F. (2013). Community dynamics of coagulase-negative staphylococci during spontaneous artisan-type meat fermentations differ between smoking and moulding treatments. *Int. J. Food Microbiol.* 166 168–175. 10.1016/j.ijfoodmicro.2013.06.034 23880244

[B31] Jiménez-Colmenero F. Carballo J. Cofrades S. (2001). Healthier meat and meat products: their role as functional foods. *Meat Sci.* 59 5–13. 10.1016/s0309-1740(01)00053-5 22062500

[B32] Kumar P. Chatli M. K. Verma A. K. Mehta N. Malav O. P. Kumar D. (2017). Quality, functionality, and shelf life of fermented meat and meat products: a review. *Crit. Rev. Food Sci. Nutr.* 57 2844–2856. 10.1080/10408398.2015.1074533 26463373

[B33] Leroy F. Geyzen A. Janssens M. De Vuyst L. Scholliers P. (2013). Meat fermentation at the crossroads of innovation and tradition: a historical outlook. *Trends Food Sci. Technol.* 31 130–137. 10.1016/j.tifs.2013.03.008

[B34] Leroy F. Scholliers P. Amilien V. (2015). Elements of innovation and tradition in meat fermentation: conflicts and synergies. *Int. J. Food Microbiol.* 212 2–8. 10.1016/j.ijfoodmicro.2014.11.016 25497716

[B35] Leroy F. Verluyten J. De Vuyst L. (2006). Functional meat starter cultures for improved sausage fermentation. *Int. J. Food Microbiol.* 106 270–285. 10.1016/j.ijfoodmicro.2005.06.027 16213053

[B36] Martín A. Colín B. Aranda E. Benito M. J. Córdoba M. G. (2007). Characterization of *Micrococcaceae* isolated from Iberian dry-cured sausages. *Meat Sci.* 75 696–708. 10.1016/j.meatsci.2006.10.001 22064035

[B37] Mezzasalma V. Sandionigi A. Guzzetti L. Galimberti A. Grando M. S. Tardaguila J. (2018). Geographical and cultivar features differentiate grape microbiota in Northern Italy and Spain vineyards. *Front. Microbiol.* 9:946. 10.3389/fmicb.2018.00946 29867854 PMC5962658

[B38] Montanari C. Barbieri F. Magnani M. Grazia L. Gardini F. Tabanelli G. (2018). Phenotypic diversity of *Lactobacillus sakei* strains. *Front. Microbiol.* 9:2003. 10.3389/fmicb.2018.02003 30210476 PMC6121134

[B39] Newson R. S. Elmadfa I. Biro G. Cheng Y. Prakash V. Rust P. (2013). Barriers for progress in salt reduction in the general population. An international study. *Appetite* 71 22–31. 10.1016/j.appet.2013.07.003 23891557

[B40] Ojha K. S. Kerry J. P. Duffy G. Beresford T. Tiwari B. K. (2015). Technological advances for enhancing quality and safety of fermented meat products. *Trends Food Sci. Technol.* 44 105–116. 10.1016/j.tifs.2015.03.010

[B41] Oksanen J. Blanchet F. G. Friendly M. Kindt R. Legendre P. McGlinn D. (2019). *vegan: Community Ecology Package. R package version 2.5-4*^∗^. Available at: https://CRAN.R-project.org/package=vegan (accessed January 14, 2019).

[B42] Palla M. Cristani C. Giovannetti M. Agnolucci M. (2017). Identification and characterization of lactic acid bacteria and yeasts of PDO Tuscan bread sourdough by culture dependent and independent methods. *Int. J. Food Microbiol.* 250 19–26. 10.1016/j.ijfoodmicro.2017.03.015 28364622

[B43] Pisacane V. Callegari M. L. Puglisi E. Dallolio G. Rebecchi A. (2015). Microbial analyses of traditional Italian salami reveal microorganisms transfer from the natural casing to the meat matrix. *Int. J. Food Microbiol.* 207 57–65. 10.1016/j.ijfoodmicro.2015.04.029 26001060

[B44] R Core Team. (2018). *R: A Language and Environment for Statistical Computing.* Vienna: R Foundation for Statistical Computing.

[B45] Rantsiou K. Drosinos E. H. Gialitaki M. Urso R. Krommer J. Gasparik-Reichardt J. (2005). Molecular characterization of *Lactobacillus species* isolated from naturally fermented sausages produced in Greece, Hungary and Italy. *Food Microbiol.* 22 19–28. 10.1016/j.fm.2004.05.001 17045690

[B46] Ravyts F. De Vuyst L. Leroy F. (2012). Bacterial diversity and functionalities in food fermentations. *Eng. Life Sci.* 12 356–367. 10.1016/j.syapm.2016.09.005 27729171

[B47] Ravyts F. Steen L. Goemaere O. Paelinck H. De Vuyst L. Leroy F. (2010). The application of staphylococci with flavour-generating potential is affected by acidification in fermented dry sausages. *Food Microbiol.* 27 945–954. 10.1016/j.fm.2010.05.030 20688237

[B48] Sánchez Mainar M. Stavropoulou D. A. Leroy F. (2017). Exploring the metabolic heterogeneity of coagulase-negative staphylococci to improve the quality and safety of fermented meats: a review. *Int. J. Food Microbiol.* 247 24–37. 10.1016/j.ijfoodmicro.2016.05.021 27234590

[B49] Sawitzki M. C. Fiorentini ÂM. Bertol T. M. Sant’Anna E. S. (2009). *Lactobacillus plantarum* strains isolated from naturally fermented sausages and their technological properties for application as starter cultures. *Food Sci. Technol.* 29 340–345.

[B50] Søndergaard A. K. Stahnke L. H. (2002). Growth and aroma production by *Staphylococcus xylosus*, *S. carnosus* and *S. equorum* - a comparative study in model systems. *Int. J. Food Microbiol.* 75 99–109. 10.1016/s0168-1605(01)00729-2 11999121

[B51] Spitaels F. Wieme A. D. Janssens M. Aerts M. Daniel H.-M. Van Landschoot A. (2014). The microbial diversity of traditional spontaneously fermented lambic beer. *PLoS One* 9:e95384. 10.1371/journal.pone.0095384 24748344 PMC3991685

[B52] Stahnke L. H. Holck A. Jensen A. Nilsen A. Zanardi E. (2002). Maturity acceleration of Italian dried sausage by *Staphylococcus carnosus* - relationship between maturity and flavor compounds. *J. Food Sci.* 67 1914–1921. 10.1111/j.1365-2621.2002.tb08746.x

[B53] Stavropoulou D. A. De Vuyst L. Leroy F. (2018b). Nonconventional starter cultures of coagulase-negative staphylococci to produce animal-derived fermented foods, a SWOT analysis. *J. Appl. Microbiol.* 125 1570–1586. 10.1111/jam.14054 30053335

[B54] Stavropoulou D. A. De Maere H. Berardo A. Janssens B. Filippou P. De Vuyst L. (2018a). Species pervasiveness within the group of coagulase-negative staphylococci associated with meat fermentation is modulated by pH. *Front. Microbiol.* 9:2232. 10.3389/fmicb.2018.02232 30283431 PMC6156374

[B55] Stavropoulou D. A. Filippou P. De Smet S. De Vuyst L. Leroy F. (2018c). Effect of temperature and pH on the community dynamics of coagulase-negative staphylococci during spontaneous meat fermentation in a model system. *Food Microbiol.* 76 180–188. 10.1016/j.fm.2018.05.006 30166139

[B56] Talon R. Leroy S. Lebert I. (2007). Microbial ecosystems of traditional fermented meat products: the importance of indigenous starters. *Meat Sci.* 77 55–62. 10.1016/j.meatsci.2007.04.023 22061396

[B57] Tamang J. P. Watanabe K. Holzapfel W. H. (2016). Diversity of microorganisms in global fermented foods and beverages. *Front. Microbiol.* 7:377. 10.3389/fmicb.2016.00377 27047484 PMC4805592

[B58] Toldrá F. Reig M. (2011). Innovations for healthier processed meats. *Trends Food Sci. Technol.* 22 517–522. 10.1016/j.meatsci.2017.04.239 28494317

[B59] Urso R. Comi G. Cocolin L. (2006). Ecology of lactic acid bacteria in Italian fermented sausages: isolation, identification and molecular characterization. *Syst. Appl. Microbiol.* 29 671–680. 10.1016/j.syapm.2006.01.012 16510261

[B60] Van Kerrebroeck S. Maes D. De Vuyst L. (2017). Sourdoughs as a function of their species diversity and process conditions, a meta-analysis. *Trends Food Sci. Technol.* 68 152–159. 10.1016/j.tifs.2017.08.016

[B61] Wickham H. (2016). *ggplot2: Elegant Graphics for Data Analysis.* New York, NY: Springer-Verlag.

