# Supplementary material for: Exploring the Link Between the Geographical Origin of European Fermented Foods and the Diversity of Their Bacterial Communities: The Case of Fermented Meats
Source: Front Microbiol. 2019 Oct 9;10:2302. doi: 10.3389/fmicb.2019.02302 (PMC6794416; doi:10.3389/fmicb.2019.02302)
Supplement: Supplementary file 1 [file Data_Sheet_1.docx]

Supplementary Material

# Supplementary Figures and Tables

**Table S1.** Average MSA counts, average MRS counts and average pH and standard deviations (Sd) of samples coming from IT (Italy), ES (Spain), FR (France), BE (Belgium), and DE (Germany).

| **Sample** | **MSA counts (log(cfu/g))** | **sd** | **MRS counts (log(cfu/g))** | **sd** | **pH** | **sd** | ***Origin*** |
| --- | --- | --- | --- | --- | --- | --- | --- |
| TM2 | 6.0 | 0.2 | 7.7 | 0.3 | 5.80 | 0.15 | IT |
| TM7 | 7.8 | 0.1 | 6.7 | 0.2 | 6.04 | 0.05 | IT |
| TM10 | 4.4 | 0.9 | 8.0 | 0.1 | 5.68 | 0.01 | ES |
| TM12 | 9.5 | 0.0 | 8.1 | 0.6 | 6.94 | 0.09 | IT |
| TM13 | 7.2 | 0.7 | 8.1 | 0.2 | 5.37 | 0.04 | FR |
| TM15 | 6.8 | 0.2 | 7.7 | 0.5 | 5.69 | 0.06 | FR |
| TM16 | 4.2 | 0.1 | 5.8 | 0.8 | 5.64 | 0.06 | ES |
| TM18 | 8.4 | 0.1 | 7.5 | 1.7 | 5.25 | 0.04 | FR |
| TM19 | 8.6 | 0.0 | 8.5 | 0.3 | 5.17 | 0.04 | FR |
| TM20 | 8.3 | 0.4 | 8.9 | 0.1 | 5.50 | 0.04 | FR |
| TM21 | 6.7 | 0.2 | 8.5 | 0.0 | 5.37 | 0.03 | FR |
| TM22 | 7.6 | 0.0 | 8.6 | 0.1 | 5.29 | 0.03 | FR |
| TM23 | 7.6 | 0.0 | 8.4 | 0.1 | 5.29 | 0.10 | FR |
| TM24 | 8.1 | 0.1 | 8.7 | 0.1 | 5.38 | 0.07 | FR |
| TM25 | 9.0 | 0.1 | 8.7 | 0.1 | 5.50 | 0.10 | FR |
| TM26 | 9.1 | 0.2 | 8.3 | 0.5 | 5.42 | 0.14 | FR |
| TM27 | 9.1 | 0.0 | 8.0 | 0.3 | 5.67 | 0.05 | IT |
| TM28 | 9.1 | 0.1 | 8.5 | 0.7 | 5.56 | 0.09 | FR |
| TM29 | 8.7 | 0.1 | 7.9 | 0.4 | 5.64 | 0.02 | FR |
| TM30 | 7.1 | 0.4 | 7.9 | 0.3 | 5.35 | 0.04 | IT |
| TM31 | 5.6 | 0.1 | 7.6 | 0.4 | 4.96 | 0.00 | BE |
| TM32 | 8.2 | 0.1 | 8.7 | 0.2 | 5.48 | 0.09 | IT |
| TM33 | 7.1 | 0.3 | 8.1 | 0.2 | 5.79 | 0.08 | IT |
| TM34 | 5.5 | 0.2 | 8.2 | 0.5 | 4.84 | 0.01 | BE |
| TM35 | 6.4 | 1.6 | 7.8 | 0.2 | 4.73 | 0.01 | ES |
| TM37 | 8.5 | 0.2 | 8.4 | 0.6 | 6.05 | 0.06 | IT |
| TM39 | 8.9 | 0.9 | 8.8 | 0.1 | 5.81 | 0.13 | FR |
| TM41 | 6.7 | 0.1 | 8.1 | 0.3 | 5.20 | 0.03 | FR |
| TM44 | 4.7 | 0.3 | 5.2 | 0.2 | 5.91 | 0.03 | ES |
| TM45 | 8.0 | 0.3 | 8.3 | 0.2 | 5.93 | 0.06 | FR |
| TM46 | 5.0 | 0.3 | 8.6 | 0.1 | 4.76 | 0.01 | BE |
| TM47 | 6.9 | 0.1 | 7.6 | 0.1 | 6.37 | 0.02 | IT |
| TM50 | 6.3 | 0.0 | 8.2 | 0.1 | 5.40 | 0.05 | ES |
| TM52 | 5.8 | 0.6 | 7.6 | 0.1 | 5.63 | 0.01 | FR |
| TM54 | 5.9 | 0.2 | 8.3 | 0.3 | 4.67 | 0.04 | ES |
| TM55 | 7.0 | 0.1 | 8.1 | 0.2 | 5.46 | 0.06 | FR |
| TM56 | 7.1 | 0.0 | 8.7 | 0.3 | 5.90 | 0.02 | ES |
| TM57 | 6.6 | 0.2 | 9.1 | 0.1 | 5.80 | 0.04 | ES |
| TM58 | 5.7 | 0.2 | 7.4 | 0.4 | 5.09 | 0.02 | FR |
| TM59 | 5.6 | 0.1 | 7.6 | 0.7 | 5.47 | 0.11 | ES |
| TM60 | 5.6 | 0.2 | 7.9 | 0.2 | 4.85 | 0.01 | DE |
| TM61 | 7.3 | 0.1 | 7.9 | 0.2 | 5.59 | 0.04 | IT |
| TM62 | 4.5 | 0.0 | 8.8 | 0.1 | 4.80 | 0.01 | DE |
| TM63 | 6.2 | 0.3 | 8.2 | 0.3 | 5.21 | 0.01 | IT |
| TM64 | 5.6 | 0.3 | 8.2 | 0.2 | 5.05 | 0.01 | BE |
| TM65 | 4.7 | 0.5 | 7.1 | 0.4 | 5.06 | 0.01 | FR |
| TM66 | 6.2 | 0.2 | 8.1 | 0.0 | 5.19 | 0.01 | FR |
| TM67 | 5.4 | 1.3 | 8.2 | 0.2 | 4.85 | 0.03 | BE |
| TM69 | 6.3 | 0.1 | 7.7 | 0.4 | 5.07 | 0.04 | FR |
| TM70 | 5.8 | 0.5 | 8.1 | 0.4 | 4.87 | 0.00 | BE |
| TM71 | 5.1 | 0.4 | 7.7 | 0.4 | 5.01 | 0.01 | BE |
| TM72 | 5.6 | 0.0 | 8.0 | 0.1 | 4.87 | 0.01 | BE |
| TM73 | 5.5 | 0.3 | 8.3 | 0.2 | 4.97 | 0.01 | BE |
| TM74 | 6.5 | 0.2 | 8.1 | 0.5 | 5.32 | 0.02 | BE |
| TM76 | 5.2 | 0.5 | 8.0 | 0.1 | 4.65 | 0.01 | DE |
| TM77 | 5.5 | 0.2 | 8.0 | 0.2 | 4.76 | 0.00 | BE |
| TM79 | 5.6 | 0.2 | 7.3 | 0.1 | 5.20 | 0.02 | IT |
| TM80 | 7.3 | 0.0 | 8.1 | 0.2 | 5.16 | 0.02 | FR |
| TM81 | 5.6 | 0.3 | 7.5 | 0.4 | 4.79 | 0.01 | DE |
| TM82 | 5.5 | 0.3 | 8.3 | 0.2 | 4.95 | 0.00 | BE |
| TM83 | 5.7 | 0.1 | 7.0 | 0.3 | 5.29 | 0.02 | IT |
| TM84 | 6.4 | 0.1 | 8.7 | 0.0 | 4.84 | 0.02 | ES |
| TM85 | 6.8 | 0.2 | 7.9 | 0.0 | 5.38 | 0.05 | ES |
| TM86 | 6.7 | 0.1 | 8.5 | 0.3 | 5.06 | 0.02 | IT |
| TM87 | 6.1 | 0.2 | 7.8 | 0.3 | 6.00 | 0.04 | IT |
| TM88 | 4.8 | 0.5 | 7.9 | 0.1 | 5.80 | 0.00 | ES |
| TM89 | 8.2 | 0.2 | 8.8 | 0.1 | 5.64 | 0.03 | IT |
| TM90 | 6.8 | 0.2 | 8.0 | 0.0 | 5.40 | 0.01 | FR |
| TM91 | 6.1 | 0.1 | 7.5 | 0.2 | 6.06 | 0.02 | ES |
| TM92 | 8.5 | 0.1 | 7.9 | 0.2 | 5.51 | 0.02 | FR |
| TM93 | 7.4 | 0.0 | 7.9 | 0.2 | 5.62 | 0.11 | IT |
| TM95 | 7.8 | 0.1 | 8.8 | 0.1 | 5.67 | 0.01 | FR |
| TM96 | 7.1 | 0.1 | 8.2 | 0.1 | 5.49 | 0.04 | FR |
| TM98 | 7.0 | 0.1 | 8.2 | 0.2 | 5.54 | 0.01 | FR |
| TM99 | 7.3 | 0.3 | 7.9 | 0.2 | 6.05 | 0.02 | FR |
| TM100 | 7.9 | 0.4 | 7.9 | 0.3 | 5.38 | 0.06 | FR |
| TM101 | 7.4 | 0.6 | 7.4 | 0.1 | 5.60 | 0.06 | FR |
| TM102 | 6.7 | 0.1 | 8.0 | 0.1 | 5.07 | 0.08 | FR |
| TM103 | 6.9 | 0.2 | 8.2 | 0.2 | 5.49 | 0.05 | ES |
| TM104 | 7.3 | 0.1 | 7.6 | 0.4 | 5.81 | 0.05 | ES |

**
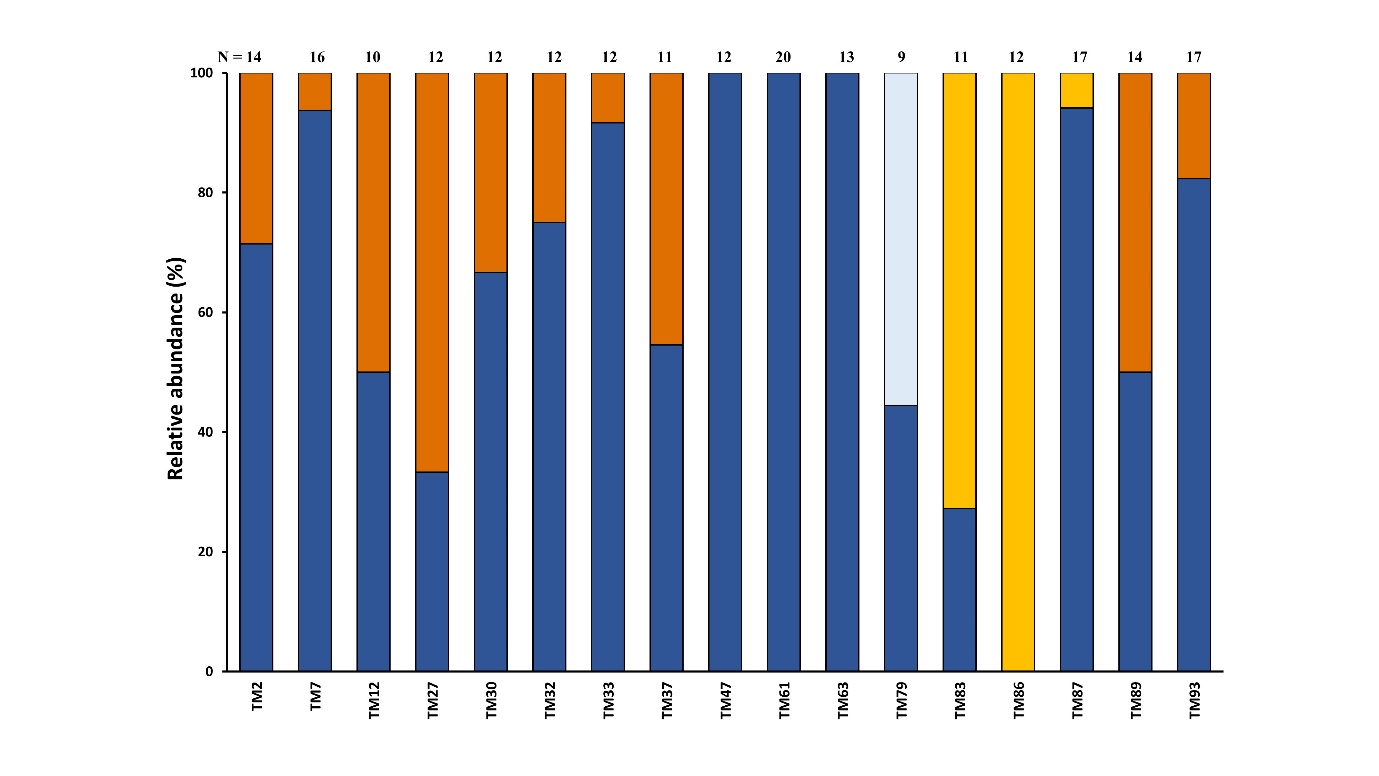
**

**Figure S1.** The relative abundance of *Staphylococcus carnosus* ()*, S. equorum* ()*, S. saprophyticus* ()*,*  *S. succinus* () and *S. xylosus* () in fermented meat products from Italy. Total number of isolates (N) obtained from MSA per sample is displayed above each bar.


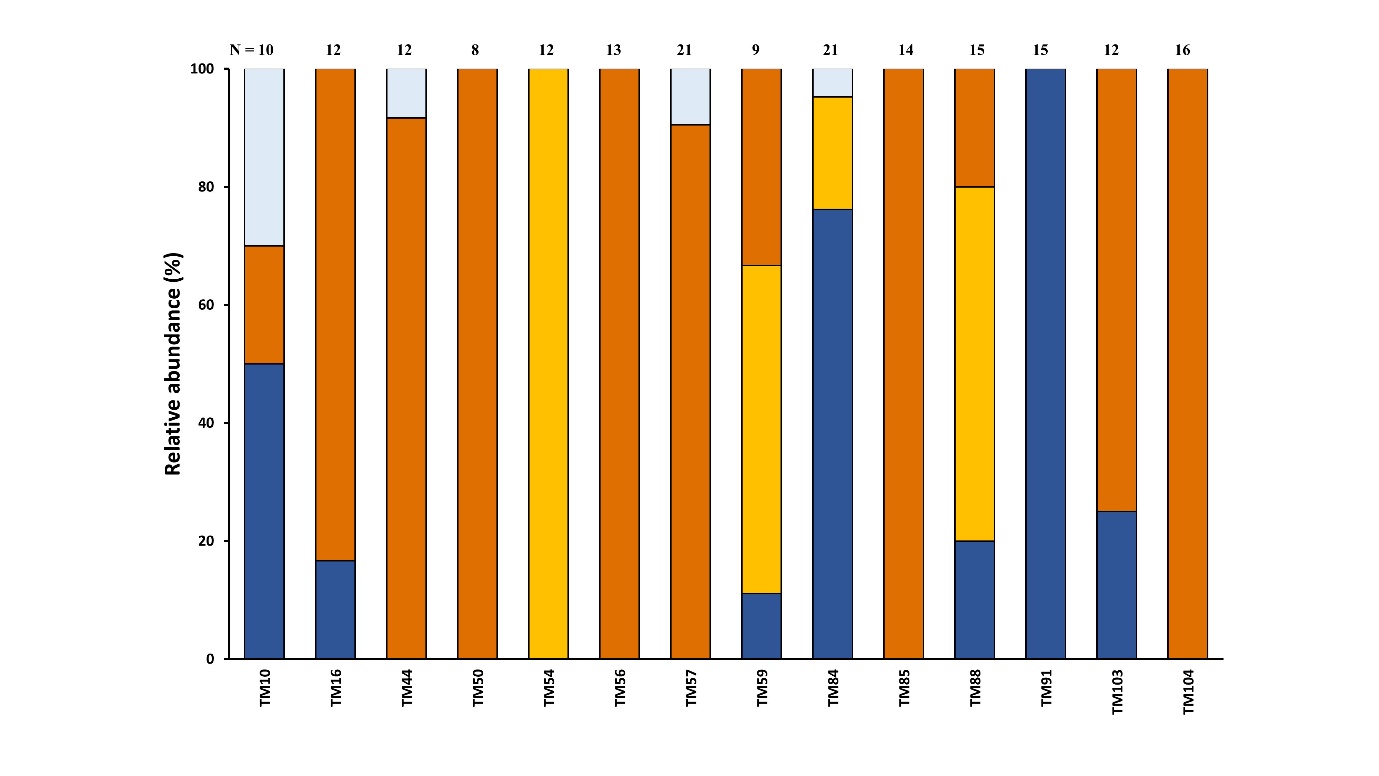


**Figure S2.** The relative abundance of *Staphylococcus carnosus* ()*, S. equorum* ()*, S. saprophyticus* ()*,*  *S. succinus* () and *S. xylosus* () in fermented meat products from Spain. Total number of isolates (N) obtained from MSA per sample is displayed above each bar.


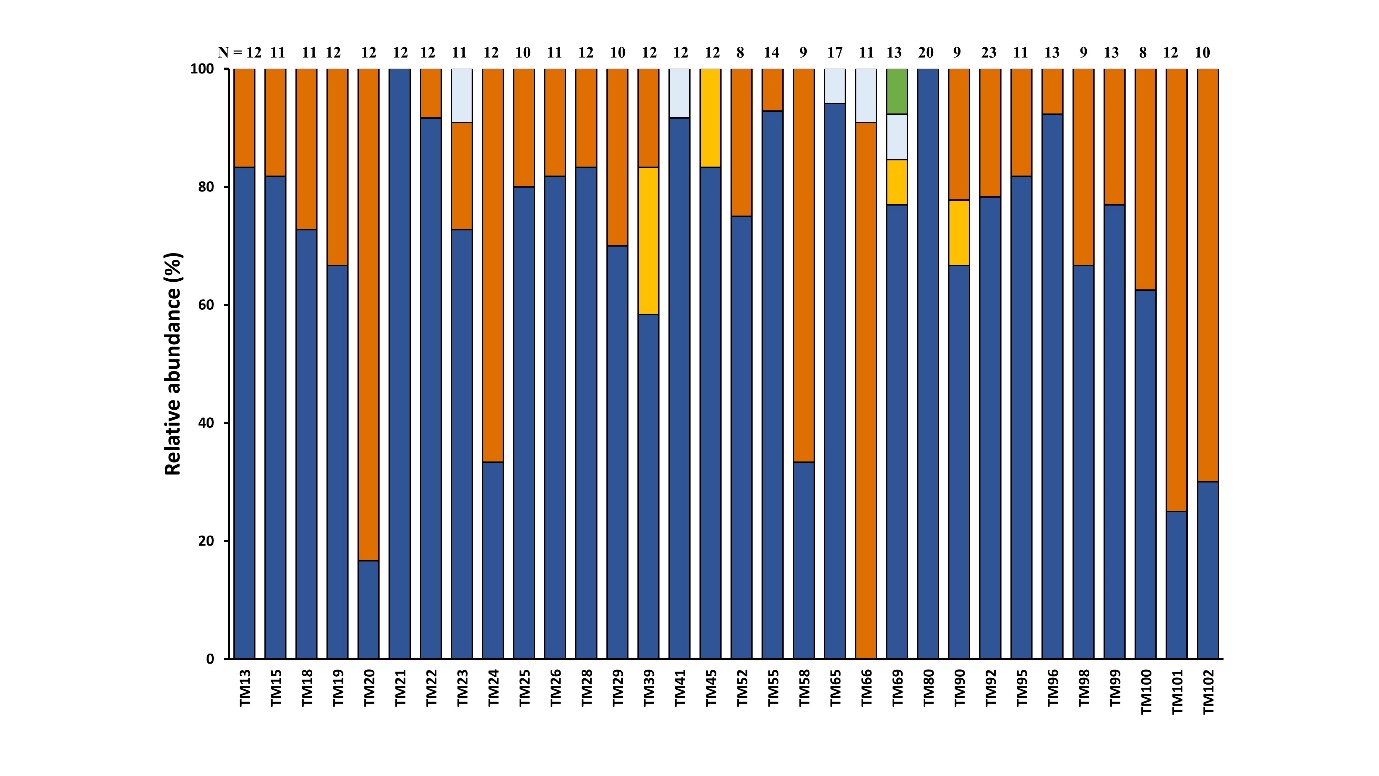


**Figure S3.** The relative abundance *Staphylococcus carnosus* ()*, S. equorum* ()*, S. saprophyticus* ()*,*  *S. succinus* () and *S. xylosus* () in fermented meat products from France. Total number of isolates (N) obtained from MSA per sample is displayed above each bar.


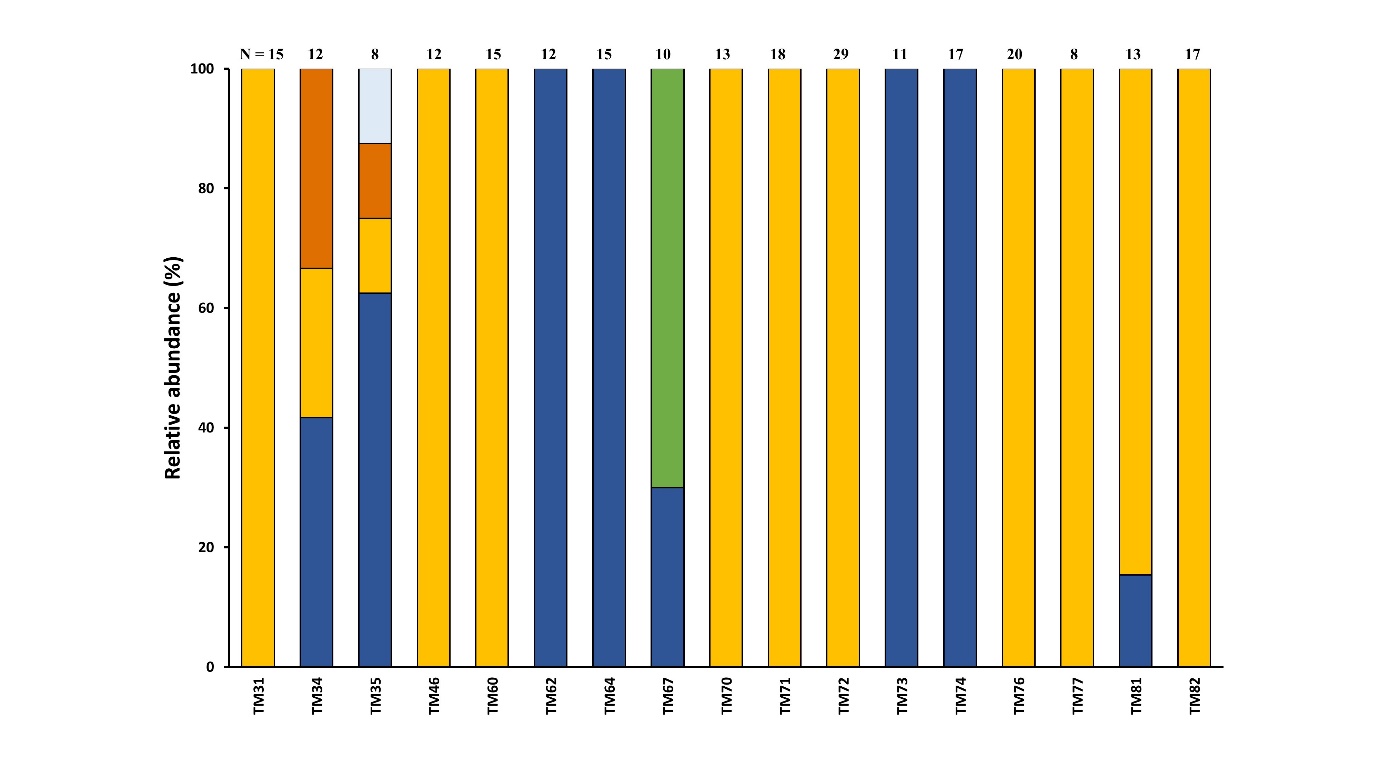


**Figure S4.** The relative abundance of *Staphylococcus carnosus* ()*, S. equorum* ()*, S. saprophyticus* ()*,*  *S. succinus* () and *S. xylosus* () in fermented meat products from Belgium and Germany. Total number of isolates (N) obtained from MSA per sample is displayed above each bar.


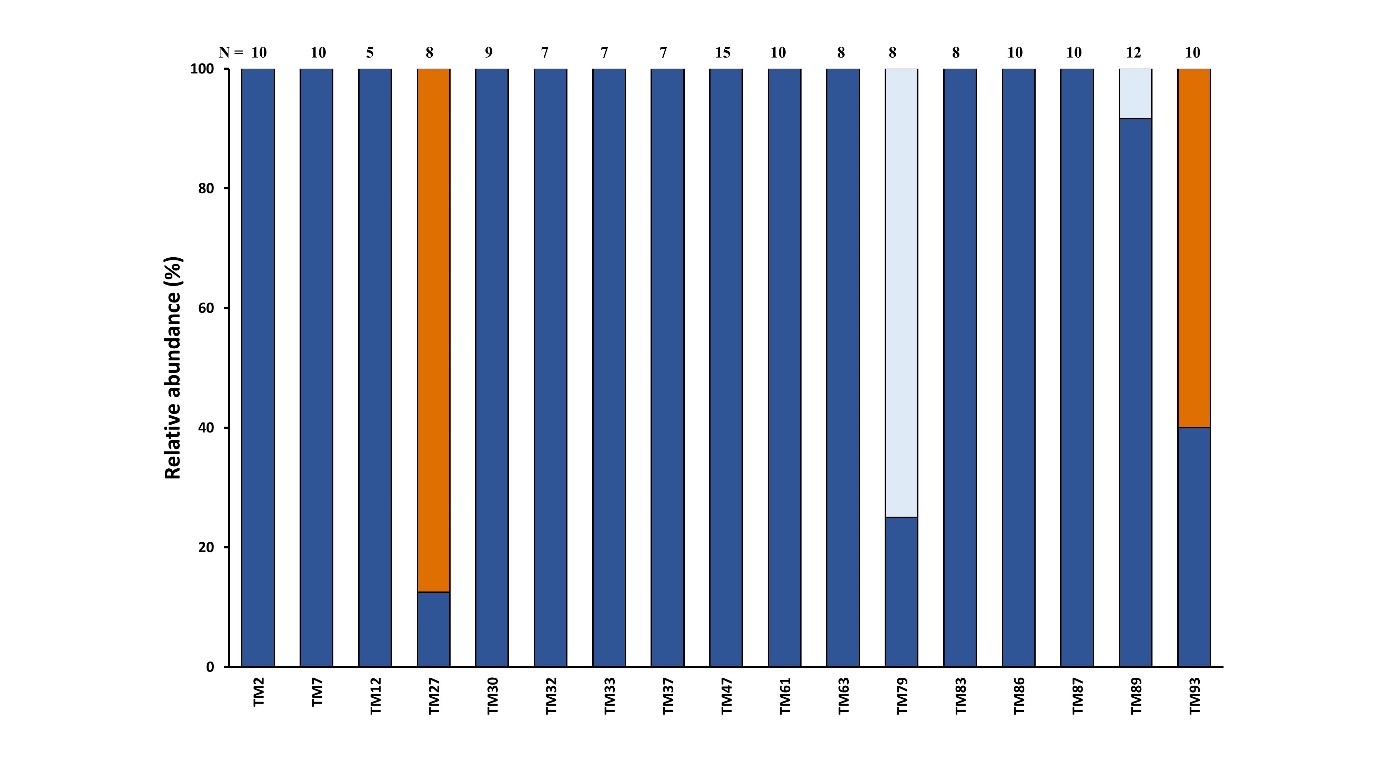


**Figure S5.** The relative abundance of *Lactobacillus alimentarius* (), *L. curvatus* (), *L. plantarum* (), *L. sakei* () and *Pediococcus pentosaceus* () in fermented meat products from Italy. Total number of isolates (N) obtained from MRS agar per sample is displayed above each bar.


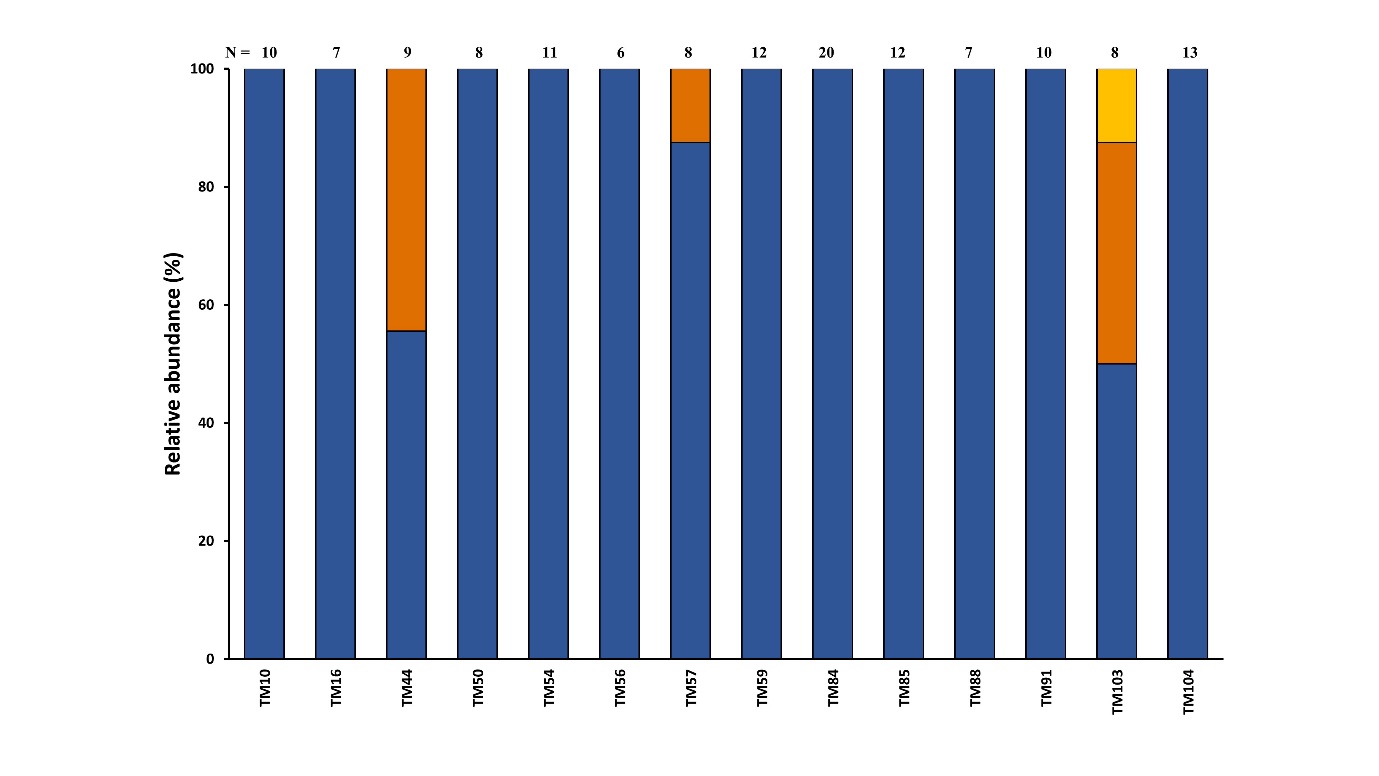


**Figure S6.** The relative abundance of *Lactobacillus alimentarius* (), *L. curvatus* (), *L. plantarum* (), *L. sakei* () and *Pediococcus pentosaceus* () in fermented meat products from Spain. Total number of isolates (N) obtained from MRS agar per sample is displayed above each bar.

**
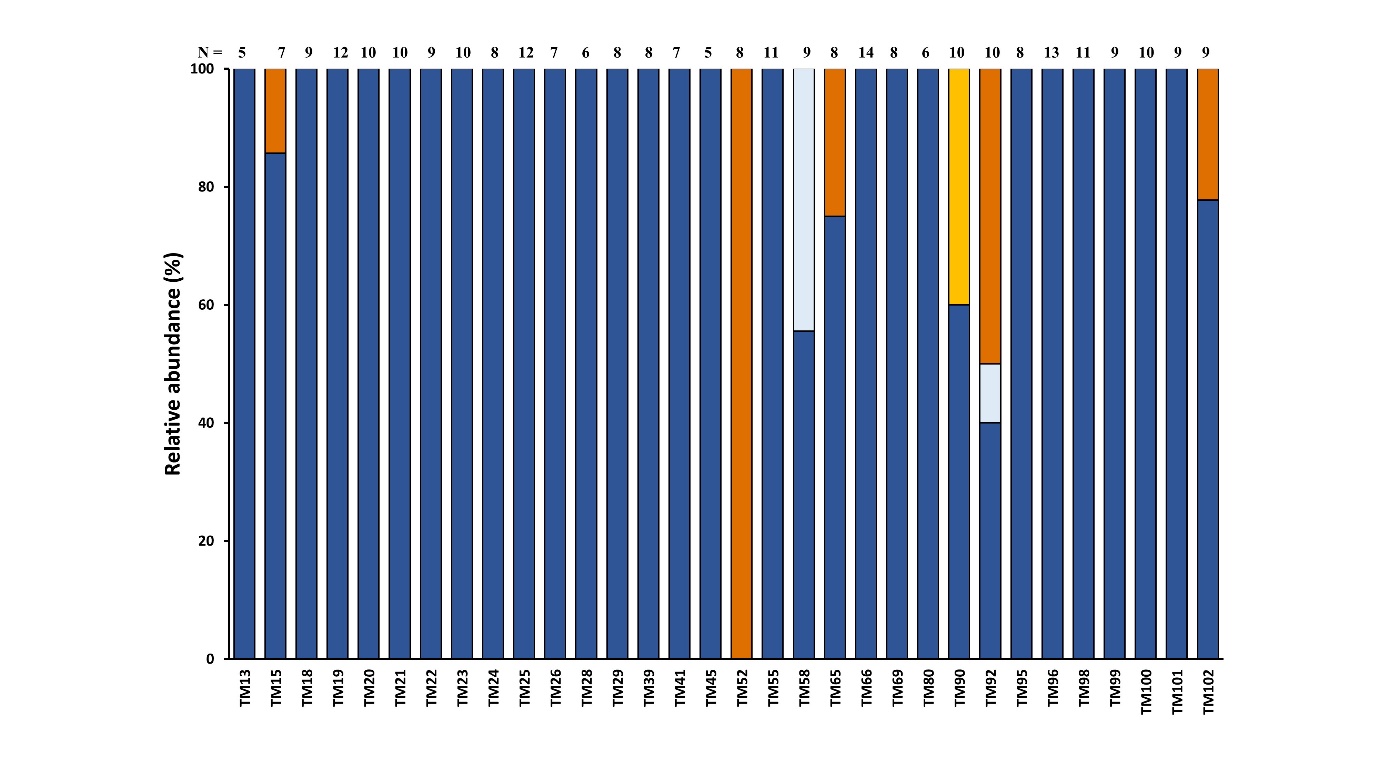
**

**Figure S7.** The relative abundance of *Lactobacillus alimentarius* (), *L. curvatus* (), *L. plantarum* (), *L. sakei* () and *Pediococcus pentosaceus* () in fermented meat products from France. Total number of isolates (N) obtained from MRS agar per sample is displayed above each bar.


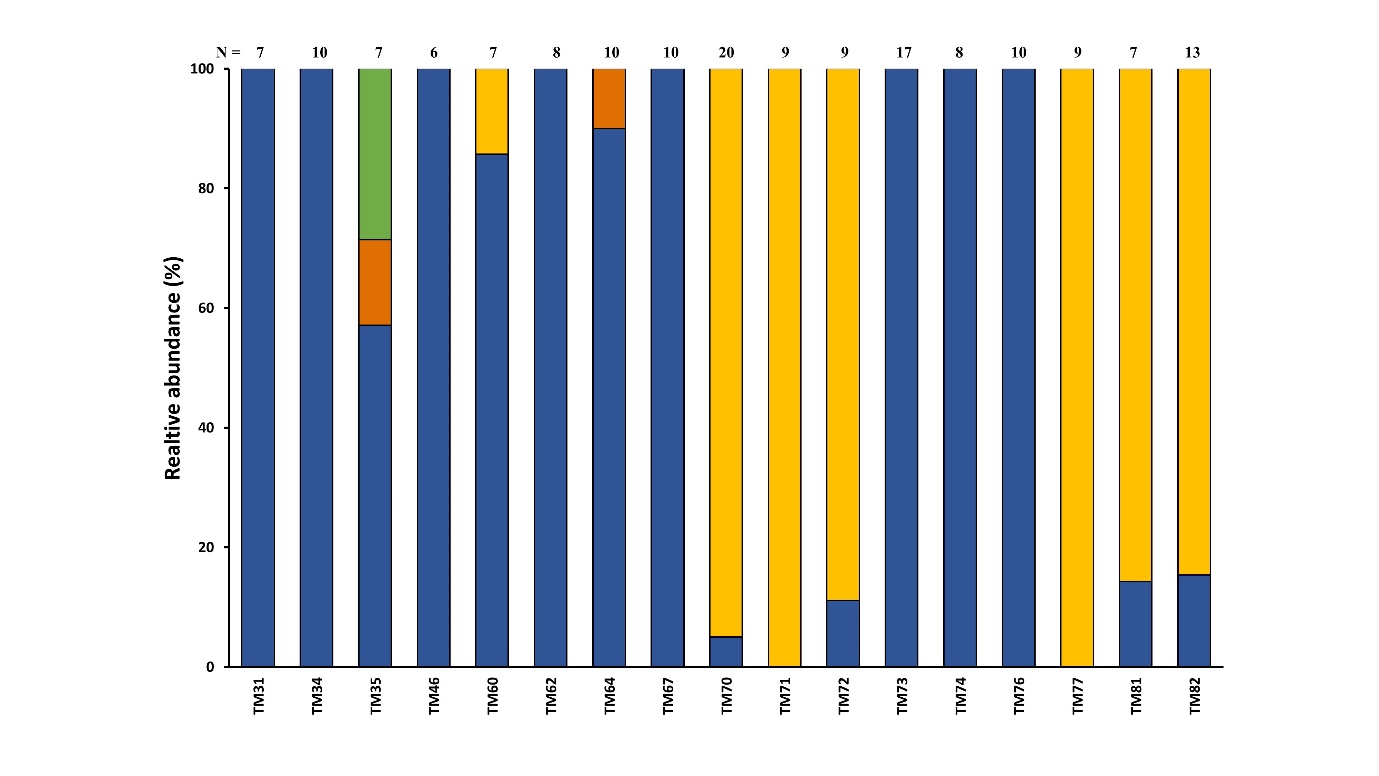


**Figure S8.** The relative abundance *Lactobacillus alimentarius* (), *L. curvatus* (), *L. plantarum* (), *L. sakei* () and *Pediococcus pentosaceus* () in fermented meat products from Belgium and Germany. Total number of isolates (N) obtained from MRS agar per sample is displayed above each bar.
